# A Case of Endemic Syphilis, Iran

**DOI:** 10.3201/eid1901.120756

**Published:** 2013-01

**Authors:** Alireza Abdolrasouli, Adam Croucher, Yahya Hemmati, David Mabey

**Affiliations:** Author affiliations: Imperial College Healthcare National Health Service Trust, London, UK (A. Abdolrasouli, A. Croucher);; Imperial College London, London (A. Abdolrasouli);; Marie-Curie Medical Institute, Tehran, Iran (A. Abdolrasouli, Y. Hemmati);; London School of Hygiene and Tropical Medicine, London (D. Mabey)

**Keywords:** endemic syphilis, treponematosis, syphilis, pinta, bejel, yaws, Treponema pallidum endemicum, bacteria, Iran, nonvenereal syphilis, nonvenereal treponematoses, azithromycin, penicillin, treponemal spirochete

**To the Editor:** Endemic syphilis, also known as bejel, is a nonvenereal treponematosis with onset in early childhood; the disease is caused by the bacterium *Treponema pallidum* subsp. *endemicum*. Until the 1970s, the disease was endemic to many parts of the world, including the Middle East; aggressive treatment programs abated its prevalence, but such programs have since ceased. Transmission occurs through contact with infectious lesions on the skin and mucous membranes and with contaminated drinking vessels (1). We report a case of bejel in a young boy in Iran, manifested by gummatous ulcerating lesions of the face.

In November 2010, a 14-year-old Iranian boy was brought by his grandfather to our private infectious diseases clinic in Tehran, Iran, because of cutaneous lesions on his face, which had increased progressively over 9 months. This adolescent had spent his childhood in Izeh, in the southwest region of Iran. He had 5 healthy siblings, and the family medical history was unremarkable. He reported experiencing a rash in childhood without mucous membrane involvement but had no history of joint or bone pain.

Examination revealed disfiguring gummatous lesions infiltrating the skin of the nose, glabella, and forehead, with clustered nodules in the left interciliary region (Figure). No other abnormality was found. He denied any sexual contacts, and there were no stigmata of congenital syphilis. Skin biopsy was refused. Tuberculin skin test results were negative. Full blood count, erythrocyte sedimentation rate, and C-reactive protein level were within reference ranges. The Venereal Disease Research Laboratory test result was positive (titer >640), and a fluorescent treponemal antibody absorption test result was strongly reactive. Because of the positive serologic test results and a preliminary diagnosis of benign tertiary syphilis, the patient was treated with 2.4 million units of benzathine penicillin G, by intramuscular injection, once per week for 3 weeks. The ulcerations completely resolved, and an atropic scar and peripheral hyperpigmentation developed over the 3-week period. The patient did not return for follow-up examination.

**Figure:**
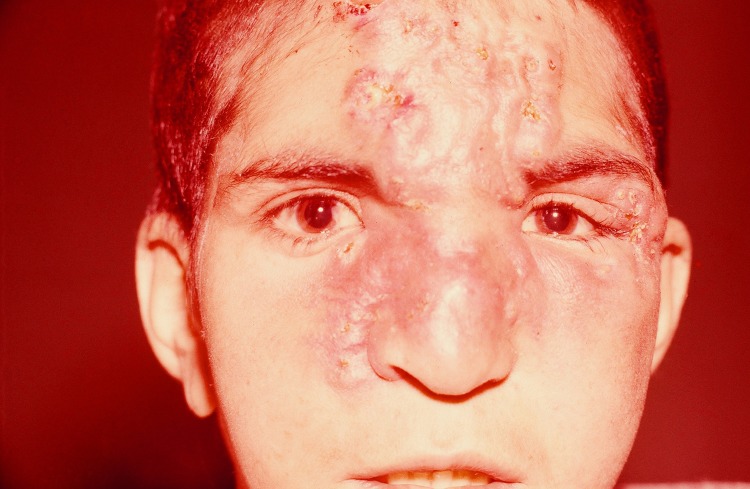
Disfiguring infiltration of the nose, glabella, and forehead with clustered nodules in left interciliary region of boy with endemic syphilis, Iran, 2010.

Serologic tests cannot distinguish between bejel and venereal syphilis. For this patient, lack of history of primary chancre, absence of cardiovascular and neurologic complications in the chronic stage of the infection, absence of history of any sexual activity, and socioeconomic background are suggestive of the nonvenereal subspecies. Because the boy had no syphilitic stigmata and his siblings were healthy, congenital syphilis is unlikely.

By the early 1970s, the global prevalence of endemic treponematoses (pinta, bejel, and yaws) had been reduced from 50 million to 2.5 million cases because of widespread use of long-acting, injectable penicillin in the 1950s and 1960s, led by the World Health Organization (WHO) and the United Nations Children’s Fund (2). However, penicillin mass treatment campaigns were not maintained and, as a result, the disease has reemerged. In 1995, WHO estimated the total number of treponematoses cases (infectious, latent, and late-stage) to be 2.6 million worldwide, including 460,000 infectious cases (3). Most of these were cases of yaws in Africa and Southeast Asia.

Bejel predominantly affects children <15 years of age. Poor personal hygiene and overcrowding facilitate transmission of infection (1,4). Manifestation as primary lesions is rare; secondary lesions or rashes are common and are usually succeeded by a period of latency. Angular stomatitis, papules, mucous patches, and macules on the moist areas of the body are the most typical manifestations. Condylomata lata, similar to those seen in venereal syphilis and yaws, can occur. If late-stage disease develops, it usually affects the skin, the long bones of the legs, and the cartilage. Cartilage damage may result in severe destruction of the nose and palate (gangosa). Whether bejel is transmitted congenitally is unknown (1,4).

Bejel was known to be endemic to the Middle East and was prevalent in Iraq and in the Bedouin population in Saudi Arabia until the 1980s (5,6). In 1995, it was diagnosed in 3 children and their father in southeastern Turkey, an area where no cases of bejel had been reported for >30 years (7). In 1954, 1 epidemiologic study of bejel in Iran reported a prevalence of 23%–34% in 4 remote villages of Khousistan (8), near the home of the case-patient in the current study. Since 1954, no cases in Iran have been reported to WHO.

This case report shows that bejel continues to be transmitted among isolated, poor rural communities in Iran. Our patient was living in a remote, rural district of low socioeconomic status, and his community had almost no access to medical facilities. WHO recently convened a meeting to discuss a new initiative for the eradication of yaws, after it was demonstrated that a single oral dose of azithromycin was as effective as injected penicillin in the treatment of this disease (9,10). Bejel should be equally susceptible to eradication, but only if health services are made available to poor rural communities in areas where the disease is endemic. We recommend that countries in which this disease was formerly declared endemic initiate surveillance programs with the goal of eradication if new cases are found. 
